# Multivalent nucleosome scaffolding by bromodomain and extraterminal domain tandem bromodomains

**DOI:** 10.1016/j.jbc.2025.108289

**Published:** 2025-02-10

**Authors:** Michael D. Olp, Karina L. Bursch, Sarah L. Wynia-Smith, Raymundo Nuñez, Christopher J. Goetz, Vaughn Jackson, Brian C. Smith

**Affiliations:** 1 Department of Biochemistry, Medical College of Wisconsin, Milwaukee, Wisconsin, USA; 2 Structural Genomics Unit, Linda T. and John A. Mellowes Center for Genomic Sciences and Precision Medicine, Medical College of Wisconsin, Milwaukee, Wisconsin, USA; 3 Program in Chemical Biology, Medical College of Wisconsin, Milwaukee, Wisconsin, USA

**Keywords:** amplified luminescent proximity homogeneous assay, bioluminescence resonance energy transfer, bromodomain-containing protein 4, chromatin, computational biology, epigenetics, histone acetylation, isothermal titration calorimetry, nucleosome, sucrose gradients

## Abstract

Promoter-promoter and enhancer-promoter interactions are enriched in histone acetylation and central to chromatin organization in active genetic regions. Bromodomains are epigenetic “readers” that recognize and bind histone acetylation. Bromodomains often exist in tandem or with other reader domains. Cellular knockdown of the bromodomain and extraterminal domain (BET) protein family disrupts chromatin organization, but the mechanisms through which BET proteins preserve chromatin structure are largely unknown. We hypothesize that BET proteins maintain overall chromatin structure by employing their tandem bromodomains to multivalently scaffold acetylated nucleosomes in an intranucleosomal or internucleosomal manner. To test this hypothesis biophysically, we used small-angle X-ray scattering, electron paramagnetic resonance, and Rosetta protein modeling to show that a disordered linker separates BET tandem bromodomain acetylation binding sites by 15 to 157 Å. Most of these modeled distances are sufficient to span the length of a nucleosome (>57 Å). Focusing on the BET family member BRD4, we employed bioluminescence resonance energy transfer and isothermal titration calorimetry to show that BRD4 bromodomain binding of multiple acetylation sites on a histone tail does not increase BRD4-histone tail affinity, suggesting that BET bromodomain intranucleosome binding is not biologically relevant. Using sucrose gradients and amplified luminescent proximity homogeneous (AlphaScreen) assays, we provide the first direct biophysical evidence that BET bromodomains can scaffold multiple acetylated nucleosomes. Taken together, our results demonstrate that BET bromodomains are capable of multivalent internucleosome scaffolding *in vitro*. The knowledge gained provides implications for how BET bromodomain-mediated acetylated internucleosome scaffolding may maintain cellular chromatin interactions in active genetic regions.

Histone posttranslational modifications (PTMs) dictate transcriptional output over time and space without modifying the underlying genetic code (1). At the atomic scale, DNA is wrapped around histone octamers to form nucleosomes (2); nucleosomal histone PTMs regulate DNA accessibility in the processes of DNA replication, repair, and transcription (3, 4). On a larger scale, the nucleosome forms the functional unit of chromatin (5) that organizes eukaryotic chromatin in three-dimensions (3D). Enhancer-promoter and promoter-promoter looping interactions are especially critical for facilitating distinct 3D chromatin organization that allows enhancers and promoters to communicate within the nuclear space (6, 7, 8). These interactions often span DNA regions located hundreds of kilobases apart (6). However, the histone-binding proteins that form, maintain, and disassemble enhancer-promoter and promoter-promoter interactions are incompletely understood.

Proteins containing bromodomains, which recognize and bind lysine acetylation on histones and other nuclear proteins (9, 10, 11, 12), are candidates for regulating large-scale 3D chromatin organization. The bromodomain and extraterminal domain (BET) family of bromodomain-containing proteins [bromodomain-containing protein, testis-associated (BRDT), bromodomain-containing protein 2 (BRD2), bromodomain-containing protein 3 (BRD3), and bromodomain-containing protein 4 (BRD4)] (13, 14) has recently gained particular attention in the contexts of health and disease. Notably, BET bromodomain inhibitors have antiproliferative and antiinflammatory effects in various cellular and animal models (15, 16). Moreover, BET bromodomain inhibitors have entered clinical trials as potential treatments for various diseases, including cancer (17, 18, 19, 20) and inflammation-driven diseases (21, 22).

When considering how BET bromodomains may regulate 3D chromatin organization, the relatively weak affinity of individual bromodomains toward monoacetylated histone tail peptides *in vitro* [dissociation constants (*K*_d_) up to ∼3 mM] (9, 23) indicates that multivalency may be crucial for bromodomain binding to acetylated chromatin (24, 25). Multivalent binding modes involving individual bromodomains of the BET protein family have previously been described (9, 26, 27). For instance, the *N*-terminal bromodomains (BD1) of the BET bromodomain family bivalently bind two acetylated lysine residues in diacetylated KXXK motifs with reported *K*_d_ values as low as 7 μM (9, 26, 27, 28). Multivalent bromodomain-nucleosome interactions may also be facilitated by proteins containing multiple bromodomains in tandem (*e.g.* BRDT, BRD2, BRD3, and BRD4) (29). For example, BRD4 tandem bromodomains bind nucleosomes hyperacetylated on histones H3 (lysines 9, 14, 18, 23, and 27) and H4 (lysines 5, 8, 12, 16, and 20) with tighter affinity than nucleosomes hyperacetylated at either H3 or H4 (30). Therefore, multivalent interactions of BET proteins with acetylated lysines on both the H3 and H4 tails of the same or multiple nucleosomes are likely important for BET proteins to maintain large-scale chromatin organization. Consistent with this hypothesis, cellular BRD2 depletion results in structural disruption of chromatin boundary regions (31), BRD4 knockdown leads to widespread decompaction of subnuclear chromatin 3D structure (32), and BRDT knockout results in broad chromatin organization defects (33).

Although the histone lysine acetylation binding specificities of individual BET bromodomains have been explored in detail (9, 26, 28, 34) and the importance of BET proteins in maintaining overall chromatin structure has been identified (31, 32, 33), whether BET tandem bromodomains are capable of simultaneously binding and scaffolding multiple nucleosomes remains unknown. Such scaffolding activity, which had not been tested biophysically, has broad cellular implications for how 3D chromatin organization is established and maintained. Here, we directly test distinct mechanisms through which BET tandem bromodomains can multivalently bind and scaffold nucleosomes using biochemical, structural, biophysical, and bioinformatic techniques. We employed small-angle X-ray scattering (SAXS) and Rosetta protein modeling to demonstrate that relatively long and disordered amino-acid sequences link BET tandem bromodomains. This long and disordered linker between BET tandem bromodomains suggests they can span long-range chromatin 3D interactions. Focusing on BET family member BRD4 because of its well-known role in maintaining subnuclear chromatin 3D structure (32), we used bioluminescence resonance energy transfer (BRET) assays and isothermal titration calorimetry (ITC) to determine the distance requirements for BRD4 tandem bromodomain binding of multiple acetylation sites on one histone tail. Notably, we provide the first direct evidence for scaffolding multiple acetylated nucleosomes by BET tandem bromodomains with *in vitro* sucrose gradients and amplified luminescent proximity homogeneous assays (Alpha; AlphaScreen). Overall, our results demonstrate that BET tandem bromodomains are biophysically capable of multivalent acetylated internucleosome scaffolding. This work provides molecular underpinnings for BET bromodomain-mediated scaffolding in chromatin organization and transcriptional regulation.

## Results

### BET bromodomains are separated by flexible linkers that permit high conformational freedom

**Figure 1:**
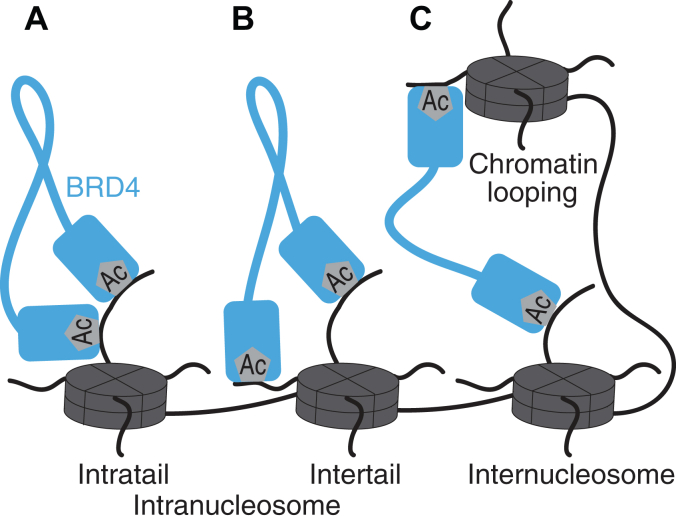
**Potential tandem bromodomain multivalent interactions with chromatin.***A* and *B*, intranucleosome interactions can occur either (*A*) between two acetylation sites on one histone tail (intratail) or (*B*) between two tails on the same nucleosome (intertail). *C*, internucleosome interactions can occur between acetylation sites on two distinct nucleosomes.

BET family members contain two tandem bromodomains (BD1 and *C*-terminal bromodomain [BD2]) at their *N* termini (Fig. S1*A*). We hypothesize that the range and occupancy of distances between tandem bromodomain acetyl-lysine binding sites dictate which multivalent nucleosome interactions are accessible to tandem bromodomains. These multivalent interactions can be classified into two types based on the distance constraints between the bromodomain acetyl-lysine binding sites: 1) multivalent bromodomain interactions within one (intratail) or between two (intertail) histone tails on one nucleosome (intranucleosome, Fig. 1, *A* and *B*) that require shorter distances between bromodomain acetyl-lysine binding sites, or 2) multivalent bromodomain interactions between histone tails on two different nucleosomes (internucleosome, Fig. 1*C*) that require longer distances between bromodomain acetyl-lysine binding sites.

**Figure 2:**
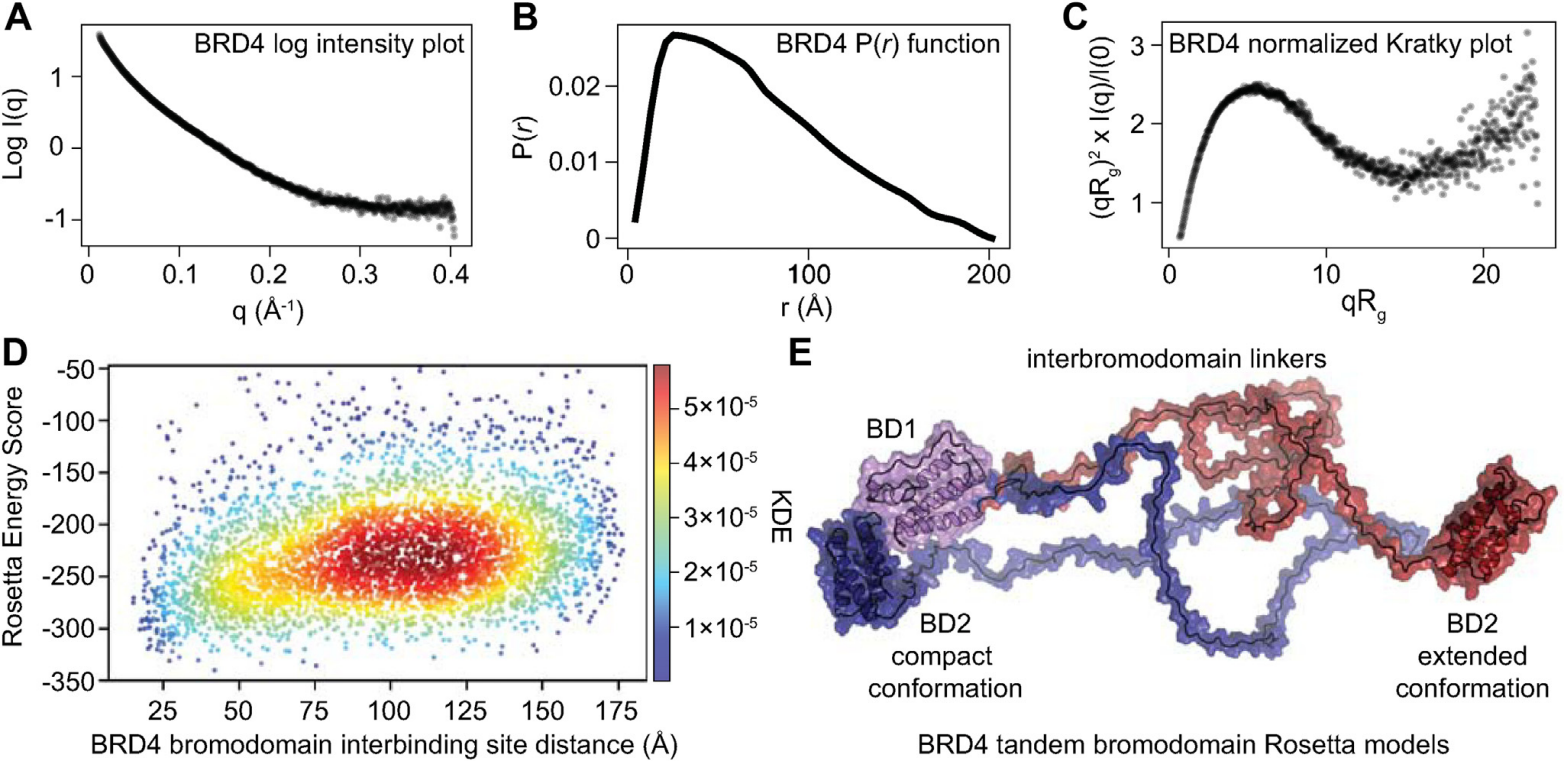
**BRD4 bromodomains are separated by flexible linkers that permit conformational freedom.***A*, log intensity plot resulting from SAXS measurements of BRD4 tandem bromodomains (aa 36–460). *B*, P(*r*) distribution resulting from SAXS measurements of BRD4 tandem bromodomains (aa 36–460). *C*, *R*_*g*_-normalized Kratky plot of BRD4 tandem bromodomain (aa 36–460) SAXS intensity. *D*, Rosetta energy score *versus* interbromodomain binding site distance plot of the top 5000 models passing the *R*_*g*_ and *D*_*max*_ constraints determined by SAXS analysis. Distances were measured using the side chain-NH_2_ groups of conserved bromodomain acetyl-lysine binding pocket asparagine residues N140 (BD1) and N433 (BD2). The color bar represents a kernel density estimation (KDE) of the distribution probability density function as calculated by the gaussian_kde method provided by the sciPy.stats Python class. *E*, representative Rosetta models demonstrating the range of interbromodomain acetyl-lysine binding site distances displayed by structures with Rosetta energy scores below the mean of the top 5000 models (−202.4). Distances between N140 of BRD4-BD1 and N433 of BRD4-BD2 range from 15.2 Å (*blue* structure) to 176.3 Å (*red* structure). BD2, *C*-terminal bromodomain; BRD4, bromodomain-containing protein 4; P(*r*), atom pair distance distribution; SAXS, small-angle X-ray scattering.

To assess how the size and conformation of all four BET proteins (Fig. S1) might impact their ability to engage in multivalent nucleosome interactions, SAXS of the BRDT/2/3/4 tandem bromodomains was performed (Figs. 2, S2–S4, and Tables S1 and S2). Atom pair distance distribution (P(*r*)) functions and Kratky representations were calculated to assess protein size/shape (35) and flexibility (36), respectively. The P(*r*) function is a histogram of the distances between every possible pair of atoms within a particle (37); globular proteins typically exhibit a bell-shaped P(*r*) curve, while disordered proteins display a less-defined curve (37). For BRD4 [amino acids (aa) 36 to 460], the P(*r*) function is asymmetric around a single peak, indicating that the BRD4 tandem bromodomains assume elongated structures with a maximum diameter (*D*_*max*_) of 201 Å (Fig. 2*B*). The Kratky plot provides a qualitative assessment of protein flexibility (36, 37). In general, scattering intensity from a compact object decreases at high scattering angles (q), producing a bell-shaped curve, while scattering intensity from a more extended object is maintained over a longer range of scattering angles, resulting in a plateau followed by a monotonic increase (36, 37). The Kratky plot for the BRD4 tandem bromodomains demonstrates an initial bell-shaped curve that increases monotonically at higher scattering angles (Fig. 2*C*). This indicates that the BRD4 tandem bromodomains contain a combination of ordered and disordered protein regions consistent with folded bromodomains connected by a flexible and disordered linker (36). Despite minimal amino acid conservancy between the linker sequences (Fig. S5), these elongated structures were conserved across other BET bromodomains, as SAXS analysis of BRDT, BRD2, and BRD3 resulted in log intensity, normalized Kratky, and P(*r*) distribution plots similar to those of BRD4 (Fig. S2). However, the interpretation of the SAXS data for BRD2 and BRD3 may be partially limited by protein aggregation, as indicated by a subset of data points lying outside the Guinier fits and the nonrandom distribution of a subset of the residuals for these proteins (Fig. S3, *C*–*F*).

When considering how tandem bromodomains engage in intranucleosome and internucleosome interactions (Fig. 1), the occupancy of distances between the acetyl-lysine binding sites of each bromodomain is particularly important. To computationally model this distance population, *ab initio* modeling of the BRD4 interbromodomain linker was performed using the Rosetta FloppyTail application (38). Output models were filtered using the SAXS experimental values (*R*_*g*_ = 55.4 ± 5.5 Å and *D*_*max*_ < 201 Å; Fig. S6) as constraints. Interbromodomain acetyl-lysine binding site distances were measured for each filtered output model using the sidechain-NH_2_ groups of the conserved Asn residues (BRD4 N140 and N433) that form a critical hydrogen bond with the acetyl-lysine oxygen in each bromodomain (9, 39, 40). While a continuous distance distribution ranging from 15 to 157 Å was observed between the two Asn residues, no Rosetta energy convergence within the allowed *R*_*g*_ and *D*_*max*_ ranges was observed (Fig. 2*D*). These results suggest there is little to no energy barrier in BRD4 tandem bromodomains to access this wide range of interbinding site distances (Fig. 2, *D* and *E*), making it possible for the BRD4 and other BET tandem bromodomains to engage in multivalent nucleosome interactions.

### Bivalent engagement of multiply acetylated histone H4 tails by BRD4 tandem bromodomains does not improve their acetylation binding affinity

Of the three possible modes of tandem bromodomain binding of acetylated nucleosomes, intratail intranucleosomal interactions (Fig. 1*A*) require the shortest distance between the two tandem bromodomain acetyl-lysine binding sites. This bivalent interaction could strengthen affinity and recruit tandem bromodomains only to chromatin regions hyperacetylated at specific lysine residues. Our SAXS-guided Rosetta modeling indicated the two BRD4 acetyl-lysine binding sites can access interbinding site distances as small as 15 Å (Fig. 2*D*), theoretically allowing bivalent interaction with two acetylation sites spaced as closely as four amino acids (41) on an extended histone tail. To investigate the ability of BRD4 tandem bromodomains to bivalently engage multiply acetylated histone tails, triacetylated histone H4 peptides harboring acetylation sites at lysine residues 5 and 8 were synthesized with a third acetylation site placed at either lysine 12, 16, or 20. We and others have shown that BRD4-BD1 preferentially binds histone H4 peptides diacetylated at lysines 5 and 8 (9, 28, 34) and hypothesized that BRD4-BD2 could simultaneously bind to the third acetylation site at lysine 12, 16, or 20 only when the distance between the lysine sites was long enough to accommodate both bromodomains.

**Figure 3:**
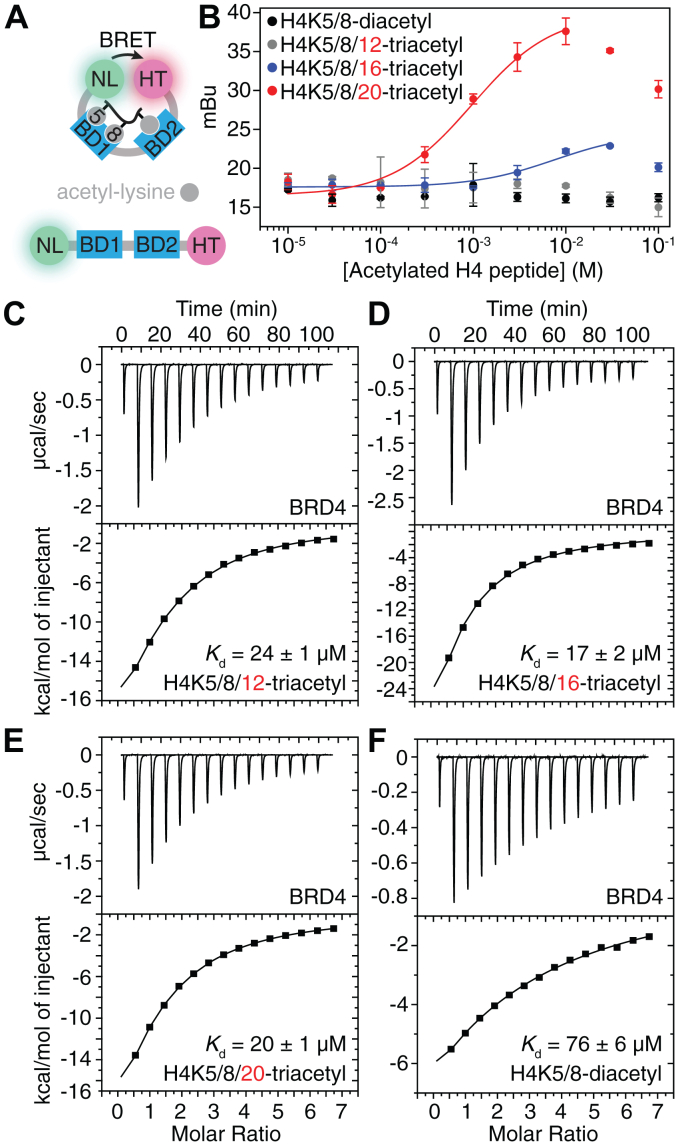
**Bivalent tandem bromodomain engagement of acetyl-lysine residues does not impact BRD4 binding affinity toward triacetylated *versus* diacetylated histone H4 tails.***A* and *B*, (*A*) nanoBRET assay schematic and (*B*) BRET signal arising from NanoLuc-BRD4-BD1_BD2-HaloTag titration with histone H4K5/8-diacetyl (*black*), H4K5/8/12-triacetyl (*gray*), H4K5/8/16-triacetyl (*blue*) and H4K5/8/20 triacetyl peptides (*red*) (n = 3), where error bars represent SD. *C*–*F*, ITC traces (n = 1) of BRD4 tandem bromodomains (aa 36–460) binding to histone (*F*) H4K5/8-diacetyl, (*C*) H4K5/8/12-triacetyl, (*D*) H4K5/8/16-triacetyl, and (*E*) H4K5/8/20-triacetyl peptides. BD1, *N*-terminal bromodomain; BD2, *C*-terminal bromodomain; BRD4, bromodomain-containing protein 4; BRET, bioluminescence resonance energy transfer; ITC, isothermal titration calorimetry; NanoLuc, nanoluciferase.

To test this hypothesis, a previously reported cellular BRET assay (42) was adapted for *in vitro* experiments using a recombinant nanoluciferase (NanoLuc)-BRD4-BD1_BD2-HaloTag (human BRD4 aa 44–460) protein expressed and purified from *Escherichia coli*. Bivalent engagement of both bromodomains in this BRD4 construct by a single ligand would bring the NanoLuc tag and fluorophore-labeled HaloTag into proximity, resulting in an increased BRET signal (Fig. 3*A*). Increased BRET signal would not be anticipated in the setting of subtle alterations in the NanoLuc tag and HaloTag relative to the protein (Fig. 3*A*), as the proximity of detection in the assay is limited to ∼5 nm (42). The addition of either the H4K5/8-diacetyl or H4K5/8/12-triacetyl peptide did not result in an increased BRET signal, consistent with monovalent binding of the BRD4 tandem bromodomains to these peptides (Fig. 3*B*). In contrast, the addition of the H4K5/8/16-triacetyl and H4K5/8/20-triacetyl peptides resulted in an increased BRET signal, with half-maximal effective concentration (EC_50_) values of 7.5 ± 3.5 μM and 0.98 ± 0.23 μM, respectively (Fig. 3*B*). The BRET signal decreased at high H4 peptide concentrations when all available acetyl-lysine sites in the peptides are saturated with monovalent bromodomain interactions, and additional acetyl-lysine sites are therefore unavailable for scaffolding (the so-called “hook effect”; Fig. 3*B*).

The increasing BRET signal in proportion to the increasing distance between the H4K5/8-diacetylated residues and the third acetyl-lysine residue (Fig. 3*B*) indicates that the greater spacing between the acetylated lysine residues of a single histone tail is structurally more permissible for bivalent occupancy by the tandem BRD4 bromodomains. Alternatively, the increased BRET signal may arise from the simultaneous binding of one BRD4 protein to H4K5/8-diacetylated residues and another BRD4 protein to either the H4K16-acetyl or the H4K20-acetyl residues on the same peptide. BRD3-BD2 also binds singly acetylated H4 lysine residues with affinities ranging from ∼10 to 150 μM (43). The results of the BRET data suggest that BRD4-BD2 may exhibit a similar binding preference. Therefore, the increased BRET signal may also correlate with the binding preference of BRD4-BD2 for acetylated lysine residues rather than the distance between the BRD4 bromodomain binding sites alone. Regardless of the differences in BRD4 bromodomain bivalent occupancy potential at different combinations of histone acetyl-lysine residues (Fig. 3*B*), the ability of the BRD4 tandem bromodomains to simultaneously bind to multiple acetyl-lysine residues on the same histone tail did not increase acetylated ligand binding affinity; all three triacetylated peptides bound to the BRD4 tandem bromodomains with approximately the same affinity (*K*_d_ = 17–24 μM, Fig. 3, *C*–*E*) as determined by ITC.

As the H4K5/8/12-triacetyl peptide did not increase the BRET signal relative to the H4K5/8-diacetyl peptide (Fig. 3*B*), yet bound to BRD4 tandem bromodomains with a comparable affinity to that of the H4K5/8/16- and H4K5/8/20-triacetyl peptides (Fig. 3, *C*–*E*), we hypothesized the 3.2 to 4.5-fold tighter BRD4 tandem bromodomain affinity toward all three triacetylated peptides relative to the H4K5/8-diacetyl peptide (*K*_d_ = 76 μM, Fig. 3*F*) is due to the increased avidity of multiple acetyl-lysine binding sites available to individual BRD4 bromodomains on a histone H4 tail (Fig. 3, *C*–*F*). To test this hypothesis, we measured ITC affinities of triacetylated histone H4 peptides toward a mutant BRD4 tandem bromodomain construct in which the conserved asparagine residue required for binding acetylated lysine residues (9, 39, 40) in BRD4-BD2 is mutated to a phenylalanine (44). Indeed, BRD4 N433F (aa 36–460) bound to H4K5/8/12-triacetyl and H4K5/8/16-triacetyl peptides with affinities (23 ± 2 and 19 ± 2 μM, respectively, Fig. S7, *A* and *B*) within error to those of the BRD4 WT construct (24 ± 1 and 17 ± 2 μM, respectively, Fig. 3, *C* and *D*). These results suggest that the increased BRD4 tandem bromodomain affinity toward triacetylated relative to diacetylated histone H4 peptides is primarily mediated by increased avidity of BRD4-BD1 toward multiple adjacent acetylated lysine residues. In addition, the WT BRD4 tandem bromodomains demonstrated relatively weak ITC affinities toward H4K5/12-diacetyl and H4K5/16-diacetyl peptides (*K*_d_ ∼200 and ∼500 μM, respectively, Fig. S7, *C* and *D*). Therefore, BRD4-BD1 binding to the H4K5/8-diacetylated histone modification is likely the primary mediator of BRD4 affinity toward histone H4 tails, while the interaction of BRD4 bromodomains with H4K16-acetylation or H4K20-acetylation plays a secondary role. Together, these studies indicate that multiple acetylations in the same histone tail do not increase BET bromodomain affinity for those acetylations. This suggests that intratail intranucleosomal engagement of multiply acetylated histone tails by BET bromodomains is unlikely to be biologically relevant for regulating chromatin architecture.

### BET tandem bromodomains engage in multivalent internucleosome interactions

Because our SAXS-guided Rosetta modeling indicated that BET bromodomains are connected by a long flexible linker (Figs. 2*E* and S5), we next investigated whether BRD4 tandem bromodomains can simultaneously bind two separate nucleosomes (Fig. 1*C*). This interaction, which represents a novel mechanism for BET bromodomain multivalent scaffolding of acetylated nucleosomes and 3D chromatin organization maintenance *via* acetylation-dependent chromatin looping, has not previously been shown experimentally. Nucleosomes measure 57 Å in length (45), and 83% of the apo BRD4 tandem bromodomain structures calculated in our SAXS-based Rosetta modeling display interbromodomain acetyl-lysine binding site distances >57 Å (Fig. 2*D*). As a result, we hypothesized that BET tandem bromodomains preferentially engage in multivalent internucleosome interactions. To experimentally measure the interbromodomain binding site distance distribution, we synthesized an electron paramagnetic resonance (EPR) probe consisting of a nitroxide spin label (TEMPO) attached to a pan-BET bromodomain inhibitor (JQ1) (13) that binds all eight acetyl-lysine binding sites of BET bromodomains with nanomolar affinity (“JQ1-TEMPO”; Fig. S8*A*). Although a BET bromodomain-targeted spin label had not been previously reported, we and others have shown that modifications at the site of JQ1 attachment to TEMPO do not affect bromodomain binding (28, 34, 46). JQ1-TEMPO binding to BRD4 tandem bromodomains was demonstrated by decreased amplitude (increased width) of the resonance lines in the continuous wave EPR spectrum (Fig. S8*B*). We attempted to collect double electron-electron resonance (DEER) measurements of JQ1-TEMPO simultaneously bound to both BRD4 acetyl-lysine binding sites to determine the native distribution of the interbromodomain binding site distances (Fig. S8*C*). However, after titrating JQ1-TEMPO to saturation, as monitored by continuous wave EPR (Fig. S8*B*), no DEER signal was detected. This result indicates that the bromodomains of the tandem BRD4 construct do not consistently adopt discrete distances required to observe unique and detectable DEER signals. Coupled with the SAXS data, this suggests that the BRD4 tandem bromodomains access a broad range of distance distributions, supporting the hypothesis that BRD4 tandem bromodomains can participate in internucleosome interactions (Fig. 1*C*).

**Figure 4:**
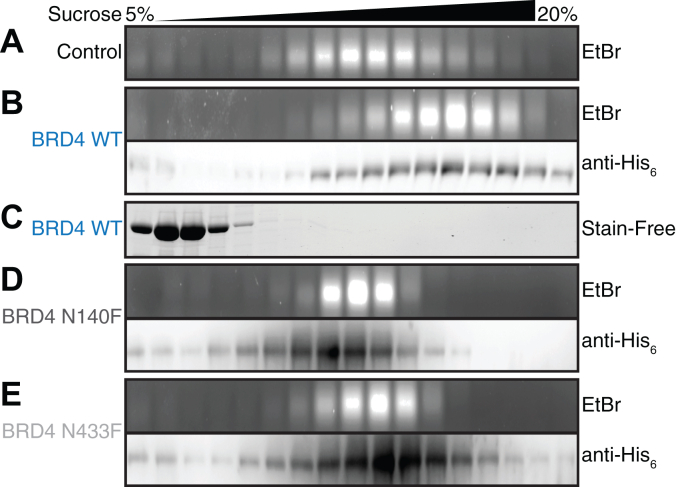
**BRD4 tandem bromodomains scaffold acetylated nucleosomes *in vitro* in sucrose gradients.***A*, control nucleosome sedimentation rate distribution in the absence of BRD4 protein constructs (representative of n = 3). *B*, WT BRD4 tandem bromodomains physically associate with calf thymus mononucleosomes and increase their sedimentation rates relative to control (representative of n = 3). *C*, WT BRD4 tandem bromodomains are associated with relatively slow sedimentation rates in the absence of nucleosomes (representative of n = 3). *D* and *E*, single bromodomain inactive mutants N140F (BRD4-BD1) and N433F (BRD4-BD2) physically associate with acetylated calf thymus mono nucleosomes but do not increase their sedimentation rate relative to control (representative of n = 2). BD1, *N*-terminal bromodomain; BD2, *C*-terminal bromodomain; BRD4, bromodomain-containing protein 4.

To directly test the ability of BRD4 tandem bromodomains to simultaneously bind separate acetylated nucleosomes, sucrose gradient binding assays were performed with BRD4 tandem bromodomains and mononucleosomes purified from calf thymus (Fig. S9). Simultaneous binding of BRD4 tandem bromodomains to multiple mononucleosomes dramatically increases their apparent size and sedimentation rate, shifting them toward an apparent size consistent with polynucleosomes in a sucrose gradient (Fig. S9). Conversely, bivalent BRD4 tandem bromodomain binding to a single mononucleosome would only modestly increase the apparent size and sedimentation rate of the mononucleosomes. To probe for BRD4 nucleosome scaffolding *in vitro*, sucrose gradient purification fractions containing only mononucleosomes were collected and applied to a second sucrose gradient either alone (Fig. 4*A*) or combined with the BRD4 tandem bromodomains (aa 36–460, Fig. 4*B*). Indeed, adding BRD4 to calf thymus mononucleosomes that natively harbor high lysine acetylation levels (47, 48) resulted in a substantially increased sedimentation rate compared to mononucleosomes applied to the gradient alone (Fig. 4, *A* and *B*). This apparent increase in nucleosome size resembling polynucleosomes indicates that BRD4 tandem bromodomains can engage in multivalent internucleosome interactions (Fig. 1*C*). In these experiments, BRD4 association with mononucleosomes was confirmed by immunoblotting for the BRD4 His_6_ tag, which displayed a correlation between BRD4 protein and mononucleosome sedimentation distributions (Fig. 4*B*). In contrast, the BRD4 tandem bromodomains remained at the top of sucrose gradients lacking mononucleosomes (Fig. 4*C*), consistent with the >4-fold higher molecular weight of mononucleosomes compared to the BRD4 tandem bromodomain construct used in these experiments. Next, we investigated whether the observed BRD4-mediated multivalent nucleosome scaffolding requires acetyl-lysine binding activity by both bromodomains. Consistent with the acetylation-binding dependence of BRD4 internucleosome interactions, no increase in nucleosome sedimentation rate was observed when either of the conserved asparagine residues required for BRD4-BD1 (N140) or BRD4-BD2 (N433) acetyl-lysine binding (9, 39, 40) were mutated to phenylalanine (44) (Fig. 4, *D* and *E*). The sedimentation rate distributions of the BRD4 mutant constructs also correlated closely with that of mononucleosomes (Figs. 4, *A*, *D* and *E*, S9), indicating that the BRD4 mutant constructs bound mononucleosomes but were incapable of multivalent nucleosome scaffolding interactions.

Additional sucrose gradient experiments were performed to determine if multivalent nucleosome scaffolding by BRD4 is generalizable to other tandem bromodomain-containing proteins within the BET family. BRDT and BRD4 have similar specificity profiles for binding acetylated histones (9), and our SAXS analysis (Figs. 2 and S2) indicated that the BRDT (aa 18–383), BRD2 (aa 71–455), and BRD3 (aa 25–416) tandem bromodomains also adopt elongated conformations in solution. Therefore, other BET proteins may also multivalently scaffold acetylated nucleosomes *in vitro*. Indeed, the BRDT tandem bromodomains increased the sedimentation of calf thymus mononucleosomes in sucrose gradients (Fig. S10), indicating that BRDT tandem bromodomains can facilitate internucleosome interactions (Fig. 1*C*). Overall, our results demonstrate that the longer flexible linker region between BET tandem bromodomains allows BET proteins to multivalently scaffold nucleosomes *in vitro* in a manner that requires histone acetyl-lysine binding by both bromodomains.

**Figure 5:**
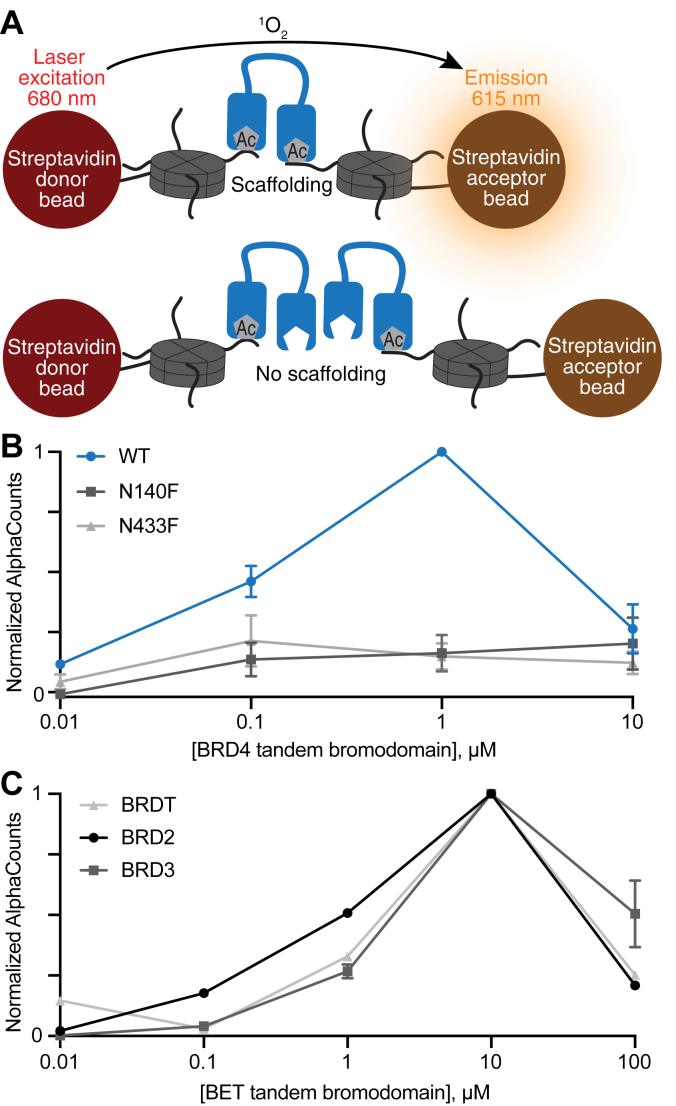
**BRD4 tandem bromodomains scaffold acetylated nucleosomes *in vitro* in AlphaScreen assays.***A*, AlphaScreen assay schematic. *B*, AlphaScreen of WT tandem BRD4 (*dark blue*), N140F (*dark gray*), or N433F (*light gray*) tandem bromodomains binding to a recombinant H4K5ac nucleosome (n = 3), where error bars represent SD. *C*, AlphaScreen of WT tandem BRD2 (*black*), BRD3 (*dark gray*), or BRDT (*light gray*) tandem bromodomains binding to a recombinant H4K5ac nucleosome (n = 3), where error bars represent SD. BRD2, bromodomain-containing protein 2; BRD3, bromodomain-containing protein 3; BRD4, bromodomain-containing protein 4; BRDT, bromodomain-containing protein, testis-associated.

To further validate the sucrose gradient results with an orthogonal biophysical assay, we developed a custom bead-based AlphaScreen assay to directly assess the ability of BET tandem bromodomains to multivalently scaffold recombinant H4K5-acetyl nucleosomes *in vitro*. In this assay, protein-mediated multivalent scaffolding of biotinylated recombinant nucleosomes attached to streptavidin-coated donor and acceptor beads brings the donor and acceptor beads into proximity, resulting in increased Alpha counts (Fig. 5*A*), which then decreases when all available acetyl-lysine sites are saturated with monovalent bromodomain interactions. Therefore, additional acetyl-lysine sites are unavailable for scaffolding (Fig. 5*A*). Consistent with the sucrose gradient experiments, BET bromodomains exhibited multivalent H4K5-acetyl internucleosome scaffolding (Fig. 5, *B* and *C*; BRD4 scaffolding activity was reduced by mutation of either conserved asparagine in the acetyl-lysine binding pockets (N140F or N433F) (Fig. 5*B*). As anticipated for scaffolding interactions, a decreased signal was observed at higher concentrations of WT BET tandem bromodomains (Fig. 5, *B* and *C*).

## Discussion

BET proteins bind to acetylated chromatin *via* their *N*-terminal bromodomains and recruit components of the transcriptional machinery *via* an extraterminal protein-protein interaction domain (49). BRD4 and BRDT also include a conserved *C*-terminal domain that interacts with the positive transcription elongation factor b (P-TEFb) and RNA polymerase II (RNAP II) during activation of transcription elongation (50, 51, 52, 53, 54). While the expression of BRDT is limited to the testes and ovaries (49), BRD4 is ubiquitously expressed (55) and widely implicated in human disease (56). Despite the biological significance of BET protein-mediated transcriptional regulation, why two tandem bromodomains are required for chromatin binding and full BET protein activity is poorly understood. Here, we investigated two potential models for the multivalent binding of BET tandem bromodomains to acetylated chromatin (Fig. 1), focusing on BRD4.

In line with other SAXS experiments (57), BRD2, BRD3, BRD4, and BRDT all demonstrate similar *R*_*g*_ (48.6–55.4 Å) and *D*_*max*_ (181–200 Å) values (Figs. 2, S2–S4, Tables S1 and S2). Consequently, our SAXS-guided Rosetta *ab initio* modeling of the BRD4 linker sequence suggests that the high degree of BRD4 interbromodomain linker flexibility is conserved across all four BET proteins. As a result, BET tandem bromodomains likely have high conformational flexibility. This permits BET proteins to engage in unique and diverse acetyl-lysine-dependent epigenetic functions through multivalent nucleosome interactions.

Consistent with the ability of bivalent BET bromodomain inhibitors to simultaneously engage both bromodomain acetyl-lysine binding sites (44, 58, 59), our SAXS-guided Rosetta modeling indicated that the BRD4 tandem bromodomains can access conformations with the two acetyl-lysine binding sites residing as little as 15 Å apart, which may permit bivalent binding of adjacent acetylation sites on individual histone tails. In our ITC studies, BRD4 binding affinity for acetylated histone ligands tightened nearly 5-fold (Fig. 3, *C*–*F*) when the number of acetyl-lysine residues on histone H4 tail peptides increased from two to three (H4K5/8-diacetyl to H4K5/8/12-triacetyl, H4K5/8/16-triacetyl, or H4K5/8/20-triacetyl). However, only the H4K5/8/16-triacetyl and H4K5/8/20-triacetyl histone peptides showed evidence of simultaneous binding to two separate bromodomains in our nanoBRET assay (Fig. 3*B*). Therefore, the observed correlation between BRD4 ligand binding affinity and the number of acetylated histone H4 tail residues likely results from the increased number of acetyl-lysine binding sites available for individual bromodomain binding rather than bivalent engagement of a single histone H4 tail peptide by a single tandem bromodomain construct (Fig. 1*A*). Alternatively, intertail interactions (Fig. 1*B*) have been shown for proteins containing a bromodomain linked to a plant homeodomain finger by a rigid linker (60, 61). However, the relatively long and flexible interbromodomain linker regions found in BET proteins (Fig. 2) suggest that BET tandem bromodomains have evolved to simultaneously engage two separate acetylated nucleosomes in a relatively unconstrained manner (Fig. 1*C*). BET tandem bromodomains may also engage in intertail interactions within the same acetylated nucleosome (Fig. 1*B*), but this possibility was not tested in this study.

The discovery of acetylated nonhistone BET bromodomain binding partners has led others to propose that the BET tandem bromodomains recruit transcription factors to chromatin regions *via* bivalent acetyl-lysine recognition. For instance, BRD4 binds acetylated versions of cyclin T1 (39, 52), the RelA/p65 subunit of NF-κB (10, 11, 62), and the transcription factor Twist (63). In addition, BET protein interactions may be more widespread than previously appreciated (57), as BET proteins have been shown to bind to numerous acetylated nuclear proteins (57). However, the ability of tandem bromodomains to directly, multivalently, and simultaneously engage separate acetylated nucleosomes had not been tested directly. Here, using sucrose gradient and AlphaScreen assays (Figs. 4, 5, and S10), we demonstrate for the first time that BET protein tandem bromodomains can scaffold separate acetylated nucleosomes and bring them into proximity (Fig. 1*C*); moreover, this nucleosome scaffolding activity is dependent on bromodomain acetyl-lysine binding (Figs. 4, *D* and *E*, 5*B*).

Hi-C chromatin conformation capture experiments have revealed that 3D chromatin organization is generally defined by combinations of cohesin-mediated CCCTC-binding factor (CTCF) loops and 40 kb-3 Mb compartmental domains not associated with CTCF peaks (64, 65). While CTCF loops are formed through CTCF- and cohesion-mediated loop extrusion (66, 67, 68, 69, 70), the formation and maintenance of phase-separated compartmental domains are associated with the chromatin transcriptional state (64, 65, 71, 72, 73). The higher-order structural organization of different chromatin regions is also associated with distinct histone PTM patterns (64). Bromodomain-mediated nucleosome scaffolding, therefore, represents a potential mechanism for the dynamic control of chromatin 3D structure *via* reversible epigenetic lysine acetylation. However, the multivalent protein-protein interactions involved in phase separation within acetylated compartmental domains are poorly understood.

**Figure 6:**
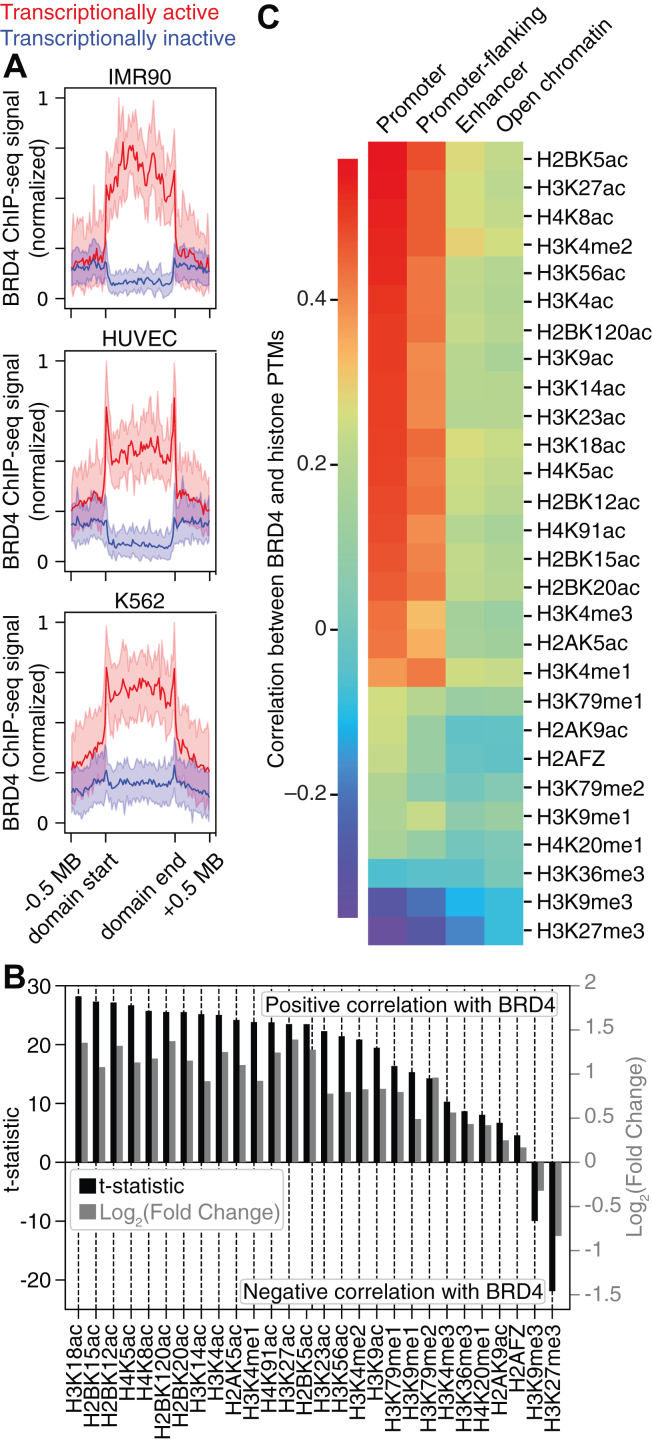
**Association between BRD4 and histone acetylation across compartmental domains may contribute to higher-order chromatin structure.***A*, normalized BRD4 ChIP-Seq profiles are enriched across transcriptionally active (*red*) relative to inactive (*blue*) compartmental domains in IMR90, HUVEC, and K562 cell lines. *B*, bar graph showing significance (two-sided *t*-statistic, *black*) and mean fold change (*gray*) of epigenetic histone PTM ChIP-Seq signal over high-*versus* low-BRD4 occupancy compartmental domains in the IMR90 cell line. *C*, Spearman correlations between ChIP-Seq signal for BRD4 and histone PTMs at Ensemble regulatory regions within compartmental domains. BRD4, bromodomain-containing protein 4; ChIP-Seq, chromatin immunoprecipitation with sequencing; PTM, posttranslational modification.

The formation of transcriptionally active compartmental domains is hypothesized to be driven by cooperative interactions between multivalent transcription factors bound to RNAP II in a “transcription factory” context (8, 74, 75, 76). Notably, BRD4 and its tandem bromodomains may be required for chromatin compartmentalization *via* liquid-liquid phase separation (77, 78, 79), as loss of BRD4 results in global chromatin decompaction in human cell lines (32). Integrating our finding that BET tandem bromodomains scaffold acetylated nucleosomes (Figs. 4, 5, and S10) *in vitro* with the knowledge that the interaction of the BRD4 *C*-terminal domain with RNAP II is crucial for the initiation of transcription elongation (53, 54), BRD4 tandem bromodomains may scaffold acetylated nucleosomes to aid the assembly and maintenance of 3D chromatin architecture at transcriptionally active compartmental domains. To provide evidence supporting this hypothesis, we conducted a bioinformatic analysis of publicly available chromatin immunoprecipitation with sequencing (ChIP-Seq) datasets deposited in the Gene Expression Omnibus and Encyclopedia of DNA Elements (ENCODE) databases (Figs. 6, S11 and S12) (64, 80, 81, 82, 83, 84). To distinguish transcriptionally active *versus* inactive compartmental domains, we used *k*-means clustering to separate previously annotated compartmental domains (GSE63525) (64) into transcriptionally active and inactive groups based on ChIP-Seq signal for H3K27ac (active) and H3K4me1 (inactive) (64) (Fig. S11). We found that BRD4 is selectively enriched throughout transcriptionally active relative to inactive compartmental domains across multiple cell lines (Fig. 6*A*), indicating that BRD4 predominantly interacts with transcriptionally active compartmental regions of chromatin.

A subset of histone PTMs [histone H2A family member Z (H2AFZ), acetylation of H3K27, H3K9, and H4K16, as well as methylation of H3K27, H3K36, H3K4, H3K79, and H4K20] have previously been used to identify and classify compartmental domains (65, 71, 73). Importantly, BRD4 bromodomains do not bind H3K27, H3K9, or H4K16 acetylation with tight affinity *in vitro* (9, 28, 34), suggesting these acetylation sites do not directly recruit BRD4 to transcriptionally active compartmental domains. Furthermore, compartmental domain distributions of H4K5 and H4K8 acetylation, the histone modifications BRD4 bromodomains bind most tightly (9, 26, 27, 28), were not previously used for compartmental domain identification. To identify histone acetylation sites correlating with BRD4 occupancy across compartmental domains, we exploited ChIP-Seq datasets for 28 distinct histone PTMs (including 17 histone acetylation sites) deposited in the ENCODE (84). ChIP-Seq signals for each histone modification were binned across IMR90 transcriptionally active and inactive compartmental domains and calculated from the mean signal per base pair over each domain. After separating IMR90 compartmental domains based on high or low BRD4 ChIP-Seq signal using *k*-means clustering (Fig. S12), we found that compartmental domains with high BRD4 occupancy were selectively enriched for histone acetylation over histone methylation and H2AFZ (Fig. 6*B*). BRD4 occupancy across compartmental domains also correlated strongly with histone acetylation sites that are known BRD4 bromodomain binding partners, including H3K14ac, H3K18ac, H4K5ac, and H4K8ac (Fig. 6*B*) (9, 28, 34, 85). Consistent with a mechanism of H3K18 acetylation driven by BRD4 chromatin binding (86, 87), H3K18ac was the most significantly enriched PTM at high BRD4 occupancy compartmental domains (Fig. 6*B*). To determine the types of regulatory chromatin regions that may participate in acetylation-dependent scaffolding by BRD4 tandem bromodomains, we used Ensembl regulatory element annotations (88) to separate compartmental domains into promoter, promoter-flanking, enhancer, and open chromatin regions. We found that correlations between BRD4 and histone acetylation were greater at promoters and promoter-flanking regions than at enhancers and open chromatin regions (Fig. 6*C*). Moreover, BRD4 bromodomain ligands H4K5ac, H5K8ac, H3K14ac, and H3K18ac (9, 28, 34, 85) were among the PTMs that highly correlated with BRD4 occupancy at promoters (Fig. 6*C*). Since BRD4 also interacts *C* terminally with RNAP II (53, 54), BRD4 tandem bromodomains may preferentially engage in multivalent scaffolding of acetylated nucleosomes at promoters to initiate transcriptional elongation from transcription factories.

In summary, our studies show that BET proteins can multivalently scaffold acetylated nucleosomes *in vitro*. We provide the first direct biophysical evidence describing internucleosomal and intratail intranucleosomal interactions between BET tandem bromodomains and chromatin. We provide a mechanistic rationale to investigate further the role of the BET family and other proteins containing linked histone-binding domains in regulating 3D chromatin organization at the cellular level. Moreover, as many human proteins encode linked diverse types of histone-binding modules beyond the tandem bromodomain-containing proteins (24), our results provide a foundation for studying the alternative tandem chromatin interaction domains that may likewise assemble and maintain higher-order chromatin structure through recognition of defined histone PTMs.

## Experimental procedures

### SAXS analysis of tandem bromodomain constructs

Briefly, 1, 2, 5, and 10 mg/ml samples of the human BRDT, BRD2, BRD3, and BRD4 tandem bromodomains were shipped to SIBYLS (89) overnight with 4 °C cold packs. SAXS data were collected *via* the mail-in program (89, 90) using the SIBYLS beamline 12.3.1 (89) at the Advanced Light Source in Lawrence Berkeley National Laboratory. The one-dimensional buffer-subtracted SAXS profile at each protein concentration was calculated from an average of 32 measurements using the SIBYLS application FrameSlice (https://sibyls.als.lbl.gov/ran). For each protein sample, the SAXS profiles at different protein concentrations were inspected to exclude the contribution from protein aggregation (caused by cumulative radiation damage) before merging to one composite SAXS profile using the SCÅTTER program (https://bl1231.als.lbl.gov/scatter/). Kratky plots, *R*_*g*_ values, *D*_*max*_, and P(*r*) functions were calculated using the SCÅTTER program, and *R*_*g*_ values for each protein were determined from the P(*r*) analysis.

### Rosetta modeling of the BRD4 interbromodomain linker

The input human BRD4 tandem bromodomains structure for *ab initio* calculations was prepared by connecting crystal structures of human BRD4-BD1 (4KV1, chain A; aa 44–168) and human BRD4-BD2 (4KV4, chain A; aa 348–459) with residues 169 to 348 built in an extended conformation. The side chains of the starting model were prepacked using the Rosetta-fixed backbone design/packing application (using the parameters -ex1, -ex2aro, use_input_sc). The FloppyTail protocol (38) was then used to generate 5000 models of the bromodomain linker in which the complete structure passed *R*_*g*_ (55.4 ± 5.5 Å) and *D*_*max*_ (<201 Å) filters determined from the SAXS analyses. The interbromodomain distances were determined by measuring the distance between the conserved Asn residue sidechain amide nitrogen atoms in BRD4-BD1 and BD2 (N140 and N433, respectively) for each model.

### NanoBRET peptide binding assays

Recombinant NanoLuc-BRD4-BD1_BD2-HaloTag (100 nM; expression and purification described in Supporting Information) with (experimental) and without (control) 100 nM HaloTag NanoBRET 618 fluorescent ligand (Promega) was combined with diacetylated or triacetylated histone peptide concentrations ranging from 10 nM to 100 μM in white, flat-bottomed 96-well plates (Corning). Plates were incubated for 30 min at 25 °C before NanoBRET Nano-Glo Substrate (Promega) was added to both control and experimental samples at a final concentration of 10 μM. Plates were read within 10 min using a Tecan Spark plate reader. A corrected BRET ratio was calculated, defined as the ratio of the emission at 610 nm/450 nm for experimental samples (*i.e.* those treated with HaloTag NanoBRET 618 fluorescent ligand) minus the emission at 610 nm/450 nm for control samples (*i.e*. those not treated with HaloTag NanoBRET 618 fluorescent ligand). BRET ratios were expressed as mBU, where one mBU corresponds to the corrected BRET ratio multiplied by 1000.

### Isothermal titration calorimetry

Binding affinities of H4(1–11)K5/8-diacetyl and H4(1–15)K5/8/12-triacetyl, H4(1–19)K5/8/16-triacetyl and H4(1–23)K5/8/20-triacetyl histone peptides toward WT or N433F BRD4 tandem bromodomains (aa 36–460) were determined using a VP-ITC instrument (MicroCal). A *C*-terminal Tyr residue was added to each peptide for concentration determination by absorbance. Briefly, 0.4 mM H4K5/8diacetyl, H4K5/8/12-triacetyl, H4K5/8/16-triacetyl, or H4K5/8/20-triacetyl peptides were injected (1 × 4 μl injection followed by 14 × 16 μl injections) into the cell containing 10 μM BRD4 (aa 36–460), and heats of binding were measured. The buffer used for ITC analysis consisted of 25 mM Hepes (pH 7.5 at 20 °C), 150 mM NaCl, and 2% v/v glycerol. Protein concentrations were determined using the method of Bradford using bovine serum albumin (BSA) as a standard (91). *K*_d_ values were determined by least-squares fitting to the raw data using Origin (OriginLab).

### Nucleosome purification

Mononucleosomes were purified as previously described (28). Briefly, 100 μl of calf thymus nuclei (10 mg/ml) in 0.25 M sucrose, 10 mM MgCl_2_, and 10 mM Tris (pH 8.0) were added to 200 μl of 100 mM NaCl, 1 mM CaCl_2_, and 40 mM Tris (pH 8.0). After 3 min equilibration at 35 °C, 2 μl of micrococcal nuclease (5 U/ml) was added, and the solution was incubated at 35 °C for 12.5 min. The nuclease reaction was then quenched with 6 μl of 250 mM EDTA, and the mixture was pelleted for 4 min at 13,200 rpm. The pellet was resuspended in 300 μl of 1 mM EDTA and pelleted for 4 min at 13,200 rpm, and 200 μl of the resulting supernatant was applied to a 4 ml sucrose gradient (5–20% w/v sucrose with 1 mM EDTA, pH 8.0) and centrifuged at 55,000*g* for 3 h. The sucrose gradient was then collected in fractions, nucleoproteins were digested with Proteinase K, and the DNA was analyzed by agarose (1.5% w/v) gel electrophoresis. Purified nucleosome concentration was determined according to DNA absorbance at 260 nm using an extinction coefficient of 6600 M^−1^ cm^−1^.

### Sucrose gradient binding assay

Subsequently, 500 nM mononucleosomes were combined with 10 μM recombinantly purified His_6_-tagged human BRDT (aa 18–383) and BRD4 (aa 36–460) WT, N140F, or N433F in 25 mM Hepes (pH 7.5 at 20 °C) and 150 mM NaCl. Each sample was incubated for 30 min at 25 °C, applied to a 4 ml sucrose gradient (5–20% w/v sucrose with 1 mM EDTA, pH 8.0), and centrifuged at 55,000*g* for 3.5 h. The sucrose gradient was then collected in fractions. Nucleosome-containing fractions were identified by agarose gel electrophoresis combined with ethidium bromide staining as described above, and BET tandem bromodomain-containing fractions were identified by anti-His_6_ tag immunoblotting or stain-free gel imaging under UV light using a ChemiDoc MP imager (Bio-Rad). Nitrocellulose membranes were first blocked using phosphate buffered saline with 0.1% v/v Tween 20 (PBST) with 3% w/v BSA. The blocked membranes were incubated with an anti-His_6_ tag primary antibody (Abgent, AM1010A) at a dilution of 1:1000 in PBST with 1.5% w/v BSA overnight at 4 °C. Membranes were then washed 3 × 5 min with PBST and incubated with a 1:10,000 dilution of goat anti-mouse IgG secondary antibody HRP (GeneTex, GTX213111–01) or goat anti-mouse-CF488A (Sigma-Aldrich, SAB4600388) in PBST with 1.5% w/v BSA for 1 to 2 h at room temperature. Finally, membranes were washed 3 × 5 min with PBST, and chemiluminescence was detected using a ChemiDoc MP imager (Bio-Rad).

### AlphaScreen

AlphaScreen assays were conducted in light gray, half-area 96-well plates (PerkinElmer, 6002350) in a total volume of 20 μl. Biotinylated recombinant H4K5ac nucleosome (EpiCypher, 16–0352) was diluted to 1.5 μM in nucleosome storage buffer (10 mM Tris–HCl, 25 mM NaCl, 1 mM EDTA, 2 mM DTT, pH 7.5), then diluted in salt-free nucleosome assay buffer (20 mM Hepes, 0.01% v/v NP-40 alternative, 0.01% w/v BSA, 1 mM DTT, pH 7.5) to create an 8 × (80 nM) nucleosome stock. Recombinantly expressed and purified tandem BET bromodomain constructs were diluted in salt-free nucleosome assay buffer to create serial 8 × (80, 8, 0.8, and 0.08 μM) protein stocks. Subsequently, 5 μl of nucleosome assay buffer (20 mM Hepes, 400 mM NaCl, 0.01% v/v NP-40 alternative, 0.01% w/v BSA, 1 mM DTT, pH 7.5) was added to each well of the plate, followed by 2.5 μl of the 8 × nucleosome stock and 2.5 μl of the 8 × protein stocks in triplicate. The plate was then wrapped in parafilm, centrifuged at 150 rcf for 30 s, and incubated for 45 min to 1 h at room temperature. A bead solution comprising 10 μg/ml streptavidin donor beads (Revvity, 6760619C) and 10 μg/ml streptavidin acceptor beads (Revvity, AL125C) was prepared in a mixture of salt-free nucleosome assay buffer and nucleosome assay buffer (final bead buffer composition 20 mM Hepes, 200 mM NaCl, 0.01% v/v NP-40 alternative, 0.01% w/v BSA, 1 mM DTT, pH 7.5). In addition, 10 μl of bead solution was added to each well under reduced light, and the plate was covered and incubated for an additional hour in the dark. Luminescence was subsequently read on a BioTek Cytation 5 imaging reader (Agilent, 16277) using the Alpha filter cube (Agilent, 1325000), and Alpha counts were analyzed using GraphPad Prism (https://www.graphpad.com/). Technical replicates of the Alpha counts for each sample were averaged, then corrected by subtracting the average signal of the experimental negative control samples (10 or 100 μM BET tandem bromodomain alone and 10 nM nucleosome alone), then normalized to the highest signal observed in the assay (1 or 10 μM).

### ChIP-Seq data analysis

To distinguish transcriptionally active *versus* inactive compartmental domains, previously annotated compartmental domains (GSE63525) (64) were separated into transcriptionally active and inactive groups based on the ChIP-Seq signal for the activating histone H3 PTMs H3K27ac and H3K4me1 (64) using *k*-means clustering. All cell lines were chosen based on the public availability of compartmental domain annotations and BRD4 ChIP-Seq datasets. BED files containing compartmental domain annotations in HUVEC, K562, and IMR90 cells were obtained as text files from GSE63525 (64). BRD4 ChIP-Seq datasets from HUVEC (GSM1305201) (83), K562 (ENCFF260JHC) (80, 81), and IMR90 (GSM1915116) (82) cells were converted from Bedgraph and Wig to BigWig format when necessary using the UCSC Genome Browser applications bedGraphToBigWig and wigToBigWig, respectively. HUVEC ChIP-Seq data was converted from the hg18 to hg19 genome assembly using the CrossMap Python package. To identify histone acetylation sites that correlate with BRD4 occupancy across compartmental domains, ChIP-Seq datasets deposited in the ENCODE (84) were analyzed for 28 distinct histone PTMs (including 17 histone acetylation sites). Histone ChIP-Seq data were obtained from ENCODE reference epigenome datasets for HUVEC (ENCSR194DQD), K562 (ENCSR612NLL), and IMR90 (ENCSR596VTT) cells as BigWig files consisting of fold-change signal over control from two merged replicates. Bigwig files were binned over compartmental domains using the DeepTools computeMatrix function in scale regions mode with a region body length of 1 Mbp and upstream and downstream distances of 0.5 Mbp. Compartmental domains were clustered based on active enhancer modification (H3K27ac and H3K4me1) or BRD4 ChIP-Seq signal using *k*-means clustering by passing the kmeans flag to the DeepTools plotProfile function. IMR90 ChIP-Seq signals for each histone modification were binned across transcriptionally active and inactive compartmental domains and calculated from the mean signal per base pair over each domain. After separating IMR90 compartmental domains based on high or low BRD4 ChIP-Seq signal using *k*-means clustering, ChIP-Seq signals across high and low BRD4 occupancy compartmental domain clusters were calculated from the mean signal per base-pair over the entire range of each domain, and significance was calculated from pooled mean signals using the ttest_ind function from the Scipy Python package assuming unequal sample variance. To determine the types of regulatory chromatin regions that may participate in acetylation-dependent scaffolding by the BRD4 tandem bromodomains, compartmental domains were separated into promoter, promoter-flanking, enhancer, and open chromatin regions using Ensembl regulatory element annotations (88). Spearman correlation coefficients between ChIP-Seq signal for BRD4 and IMR90 histone marks over Ensembl regulatory features and between mean IMR90 histone mark signal per base pair over compartmental domains were calculated using the Scipy spearmanr function.

## Data availability

Except for the SAXS data, all data are contained within the article or the Supporting Information. SAXS data of BRD2, BRD3, BRD4, and BRDT tandem bromodomains have been deposited in the Small Angle Scattering Biological Data Bank (SASBDB; https://www.sasbdb.org/) with accession codes SASDK77 (BRD2, 10 mg/ml), SASDK87 (BRD2, 5 mg/ml), SASDK97 (BRD2, 2 mg/ml), SASDKA7(BRD2, 1 mg/ml), SASDKB7 (BRD3, 10 mg/ml), SASDKC7 (BRD3, 2 mg/ml), SASDKD7 (BRD3, 1 mg/ml), SASDKE7 (BRD4, 10 mg/ml), SASDKF7 (BRD4, 5 mg/ml), SASDKG7 (BRD4, 2 mg/ml), SASDKH7 (BRDT, 10 mg/ml), and SASDKJ7 (BRDT, 5 mg/ml).

## Supporting information

This article contains supporting information (91).

## Conflict of interest

The authors declare that they have no conflicts of interest with the contents of this article.
