# Supplementary material for: Multivalent nucleosome scaffolding by bromodomain and extraterminal domain tandem bromodomains[image]
Source: J Biol Chem. 2025 Feb 10;301(3):108289. doi: 10.1016/j.jbc.2025.108289 (PMC11930079; doi:10.1016/j.jbc.2025.108289)
Supplement: Supporting information [file mmc1.pdf]

Supporting Information

Multivalent nucleosome scaffolding by bromodomain and extraterminal domain tandem bromodomains

Michael D. Olp[1], Karina L. Bursch[1,2], Sarah L. Wynia-Smith[1], Raymundo Nuñez[1], Christopher J. Goetz[1], Vaughn Jackson[1], and Brian C. Smith[1,2,3*]

[1]Department of Biochemistry, Medical College of Wisconsin, Milwaukee, WI 53226, USA; [2]Structural Genomics Unit, Linda T. and John A. Mellowes Center for Genomic Sciences and Precision Medicine, Medical College of Wisconsin, Milwaukee, WI 53226, USA; [3]Program in Chemical Biology, Medical College of Wisconsin, Milwaukee, WI 53226, USA

***For correspondence:** Brian C. Smith, brismith@mcw.edu

Table of Contents

Supplementary Methods 3

Protein expression and purification. 3

Structure-based sequence alignment of the BET inter-bromodomain linkers. 3

Synthesis of JQ1-TEMPO EPR probe. 4

Electron paramagnetic resonance. 4

Supplementary Figures and Tables 5

Figure S1. Identity and purity of recombinant tandem BET bromodomain proteins. 5

Figure S2. SAXS analysis of tandem BET bromodomains other than the BRD4 tandem bromodomains. 6

Figure S3. Guinier analysis of tandem BET bromodomains. 7

Figure S4. I(0) analysis of tandem BET bromodomains. 8

Figure S5. Structure-based sequence alignment of the BET inter-bromodomain linkers. 9

Figure S6. Filtering Rosetta BRD4 tandem bromodomain models by SAXS constraints. 10

Figure S7. BRD4-BD1 binding to the H4K5/8-diacetyl histone modification is the primary mediator of BRD4 affinity toward acetylated histone H4 tails. 11

Figure S8. EPR-based strategy for BRD4 tandem bromodomain inter-acetyl-lysine binding site measurements. 12

Figure S9. Sucrose gradient purification of calf thymus nucleosomes. 13

Figure S10. Sucrose gradient binding assays of BRDT tandem bromodomains. 13

Figure S11. K-means clustering of H3K27ac and H3K4me1 ChIP-seq profiles across annotated compartmental domains (GSE63525). 14

Figure S12. Association between BRD4 ChIP-seq signal and epigenetic histone PTMs across IMR90 compartmental domains. 15

Table S1. Values calculated from SAXS analysis of tandem BET bromodomains. 16

Table S2. R_g values calculated by P(r) analysis vs. Guinier analysis of tandem BET bromodomains. 16

References 17

Supplementary Methods

Protein expression and purification. The tandem bromodomains of human BRD2 (aa 71-455), BRD3 (aa 25-416), BRD4 (aa 38-460), and BRDT (aa 18-383) were subcloned (GenScript) between the NdeI and XhoI restriction sites of a pET28b vector modified to replace the thrombin cleavage site with a tobacco etch virus (TEV) protease cleavage site between the *N*-terminal His_6 tag and the BET bromodomain protein coding region. GenScript generated BRD4 Asn mutants (N140F and N433F). NanoLuc-BRD4-BD1_BD2-HaloTag (aa 44-460) was generated (GenScript) by subcloning NanoLuc-BRD4 (aa 44-460) from the NL-BRD4 pFC27K vector (Promega) into the pH6HTC His_6HaloTag T7 vector (Promega). The recombinant His_6-tagged tandem bromodomain constructs were purified from BL21(DE3) *E. coli* using nickel affinity chromatography. Cells were transformed and grown in 2-4 L of LB in the presence of 50 μg/mL kanamycin or 100 μg/mL ampicillin to an optical density of 0.6–0.8 at 600 nm. Protein expression was induced with 0.1 mM IPTG, and the cells were shaken overnight at 18 °C. Cells from each 1 L culture were harvested by centrifugation at 5,000 × *g* and re-suspended in 30 mL of lysis buffer (50 mM HEPES, 500 mM NaCl, 5% v/v glycerol and 2.5 mM imidazole, pH 7.5) supplemented with protease inhibitors (0.3 μM aprotinin, 1 μM E-64, 1 μM leupeptin, 1 μM bestatin, 1 μM pepstatin, and 100 μM PMSF). Cells were lysed by sonication, and lysates were cleared by centrifugation for 30 min at 30,000 × *g*. The lysate supernatants were then applied to Ni-NTA resin (0.75 mL resin/L of bacterial culture) and rocked for 1 h at 4 °C. The supernatant was discarded, and the Ni-NTA resin was applied to a column and washed twice with 25 mL of lysis buffer. The protein was eluted using a step gradient of increasing concentrations of imidazole in lysis buffer (5 mL of 50, 100, 150, 200, and 250 mM imidazole). Fractions were monitored by SDS-PAGE, and those containing recombinant protein were concentrated to a volume of 1 mL and applied to a HiLoad 16/600 Superdex 75 pg column (Bio-Rad) and eluted into storage buffer (25 mM HEPES, 150 mM NaCl, 2% v/v glycerol, pH 7.5). For EPR and SAXS studies, His_6 Ni-NTA affinity tags were removed by Tobacco Etch Virus (TEV) protease cleavage before gel filtration. For all protein purifications, monomeric protein was collected based on the chromatographs resulting from size exclusion chromatography. Concentrations of purified proteins were determined by the method of Bradford using BSA as a standard[1], aliquoted, flash-frozen, and stored at -80 °C. Samples containing recombinant protein were identified by SDS-PAGE and concentrated, flash-frozen in liquid nitrogen, and stored at -80 °C until used.

Structure-based sequence alignment of the BET inter-bromodomain linkers. The canonical sequences of BRD2 (UniProt ID: P25440), BRD3 (Q15059), BRD4 (O60885), and BRDT (Q58F21) were obtained from UniProt[91] in FASTA format. UniProt IDs for each protein were submitted to the RSCB PDB[92], and the group sequence structural alignment was used to determine the amino acids at which the α-helical

secondary structure of the *N*-terminal bromodomain ended and the α-helical secondary structure of the *C*-terminal bromodomain began. The resulting defined linker sequences were submitted to the PROMALS3D[93] multiple sequence and structure alignment server for structure-based multiple sequence alignment.

Synthesis of JQ1-TEMPO EPR probe. JQ1 (5.0 mg, 12.7 µmol) was dissolved in 1 mL of DCM, 0.25 mL of TFA was added, and the reaction was stirred overnight at 25 °C to deprotect the tBu ester to the free carboxylic acid. The reaction was dried under reduced pressure, then redissolved and dried three times with 1 mL chloroform to remove residual TFA. The resulting solid was dissolved in 156.7 µL anhydrous DMF, and to the solution was added HBTU (6.22 mg, 16.4 µmol), DIEA (7.1 µL, 54.7 µmol), and 4-Amino-TEMPO (2.8 mg, 16.4 µmol). The reaction was stirred at 25 °C for 30 h and the resulting JQ1-TEMPO was purified by semipreparative HPLC on a 5 µm particle size Hypersil GOLD C18 column (ThermoFisher, 4.6 × 250 mm) using an Agilent 1100 series HPLC eluting with a gradient of 5–95% v/v acetonitrile in water with 0.1% v/v TFA. The mass of JQ1-TEMPO was confirmed by direct injection ESI mass spectrometry (QExactive, Thermo Scientific). HRMS (ESI): Exact mass calculated for $C_{28}H_{35}ClN_6O_2S$ $[M+H]^+$ 554.2231, found 554.2242.

Electron paramagnetic resonance. 75 µM BRD4 (aa 36-460) in storage buffer (25 mM HEPES, 150 mM NaCl, 25% v/v glycerol, pH 7.5) was titrated with JQ1-TEMPO to 350 µM where saturation was reached in the continuous wave (X-band) spectrum and double electron-electron resonance measurements were subsequently recorded.

Supplementary Figures and Tables

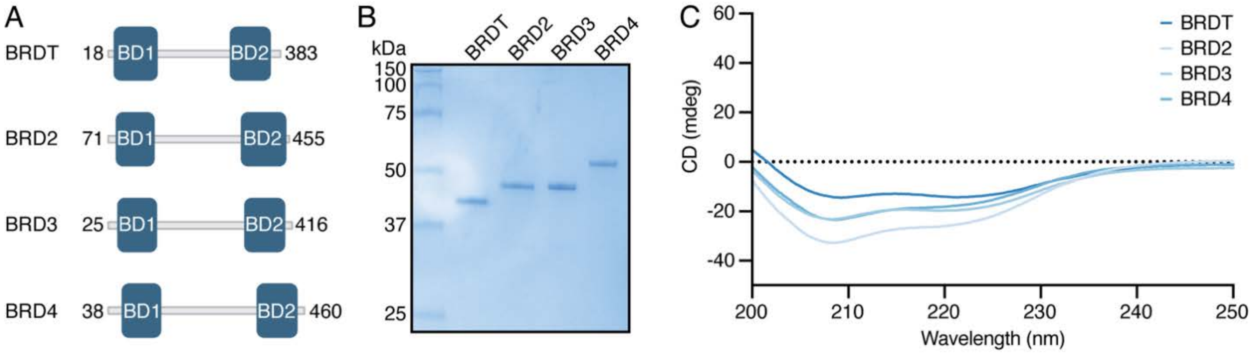

Figure S1. Identity and purity of recombinant tandem BET bromodomain proteins. **(A)** Schematic showing tandem bromodomain constructs used in this study. Domain lengths are drawn to scale. **(B)** SDS-PAGE followed by Coomassie staining of 1 µg each of His_6-tagged BRDT (44.6 kDa), BRD2 (45.9 kDa), BRD3 (46.2 kDa), and BRD4 (50.5 kDa) tandem bromodomain purification. **(C)** Circular dichroism spectra of BRDT, BRD2, BRD3, and BRD4 tandem bromodomains.

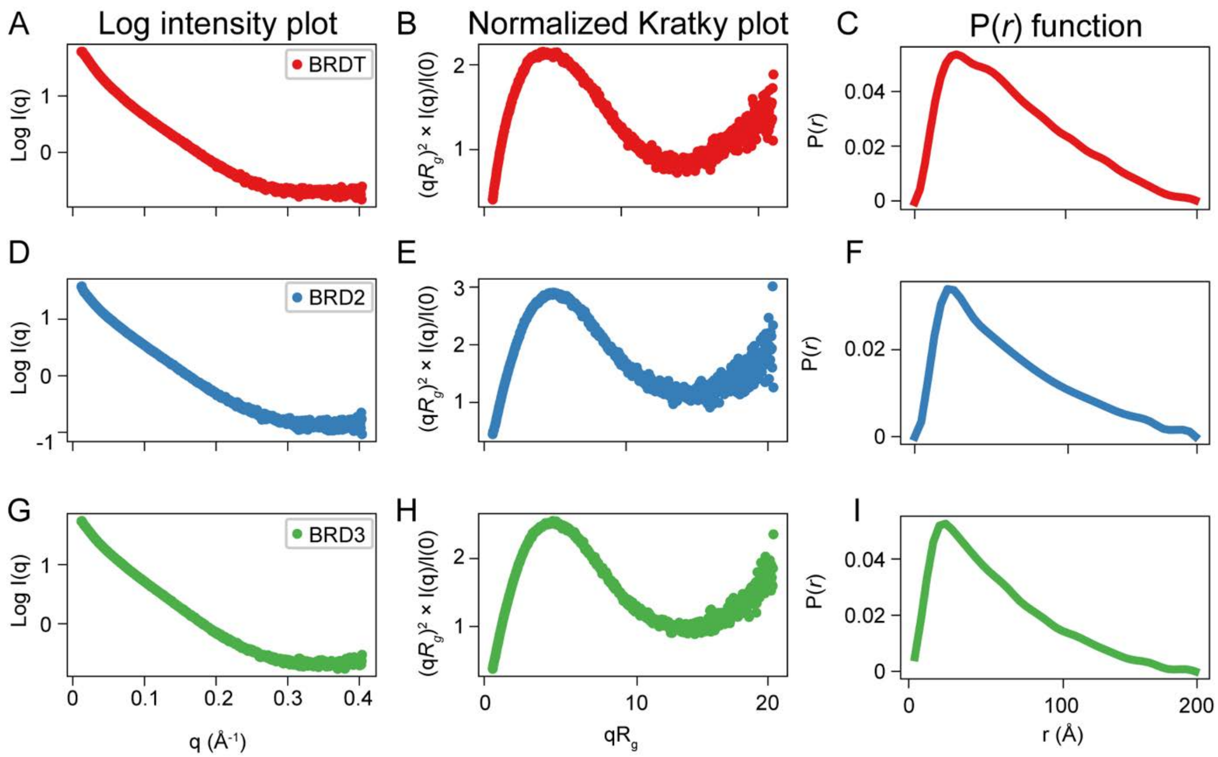

Figure S2. SAXS analysis of tandem BET bromodomains other than the BRD4 tandem bromodomains. (*See Figure 2A-C in the main text for the BRD4 tandem bromodomain data). Log intensity, R_g-normalized Kratky, and P*(r)* function plots for **(A-C)** BRDT, **(D-F)** BRD2, and **(G-I)** BRD3.

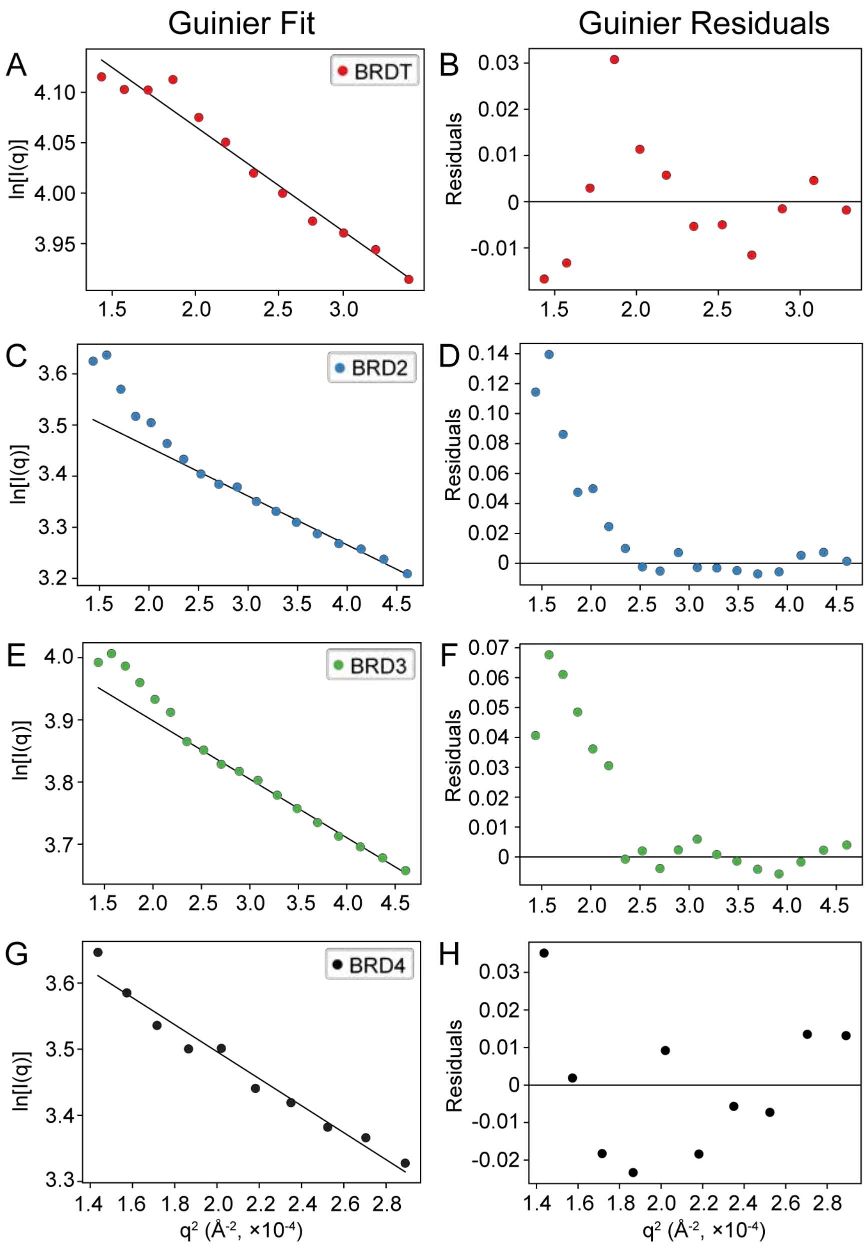

Figure S3. Guinier analysis of tandem BET bromodomains. Guinier fits and residual plots for **(A-B)** BRDT, **(C-D)** BRD2, **(E-F)** BRD3, and **(G-H)** BRD4.

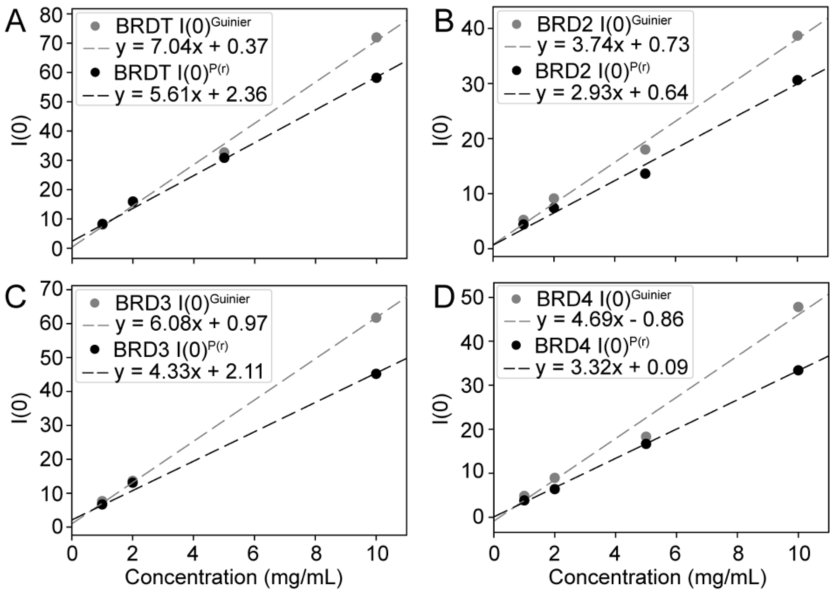

Figure S4. I(0) analysis of tandem BET bromodomains. I(0) values were obtained by both P(*r*) analysis and Guinier analysis for **(A)** BRDT, **(B)** BRD2, **(C)** BRD3, and **(D)** BRD4.

```
BRDT      133  -MPQEEQVVGVKERIKKGT----------------------QQNIAVSSAKEKSSPSATEKV--------  171
BRD2      180  -MPQEEQELVVTIPKNSHK--KGAKLAA-----LQGSVTSAHQVPAVSSVS-HTALYTPPPE--------  232
BRD3      140  -MPQEEVELLPPAPKGKGR--KPAAGAQ-----SAGT----QQVAAVSSVSPATPFQSVPPT--------  189
BRD4      163  ELPTEETEIMIVQAKGRGRGRKETGTAKPGVSTVPNT----TQAS--TPPQTQTPQPNPPPVQATPHPFP  226
Consensus      --P-EE-----------------------------------Q----------------------------

BRDT      172  ------FKQQEIPSVFPKTSISPLNVV-------------------QGASVN--SSSQTAAQVTKGVKRKAD  216
BRD2      233  ------IPTT--VLNIPHPS----VISSPLLKSLHSAG-PP-------LLAVTAAPPAQPLAKKKGVKRKAD  284
BRD3      190  ------VSQTPVIAATPVPTITANVTSVPVPPAAAPPP-PA-------TPIVPVVPPTPPVVKKKGVKRKAD  247
BRD4      227  AVTPDLIVQTPVMTVVPPQPLQ---TPPPVPPQPQPPPAPAPQPVQSHPPII--AATPQPVKTKKGVKRKAD  293
Consensus      ----------------P-----------------------------------------------KGVKRKAD

BRDT      217  TTTPATSAVKA---SSEFSPTFTEKSVALPPIKEN------MPKNVLPDSQQQYNVVKT---  266
BRD2      285  TTTPTPTAILAPGSPASPPGSLEPKAARLPPMRRESGRPIKPPRKDLPDSQQQHQSSKKGKL  346
BRD3      248  TTTPTTSAITASRSESPPPLS-DPKQAKVVARRESGGRPIKPPKKDLEDGEVPQHAGKKGKL  308
BRD4      294  TTTPTTIDPIH---EPPSLPP-EPKTTKLGQRRES-SRPVKPPKKDVPDSQQHPAPEKSS--  348
Consensus      TTTP--------------------K-----------------P-----D--------K----
```

Figure S5. Structure-based sequence alignment of the BET inter-bromodomain linkers.

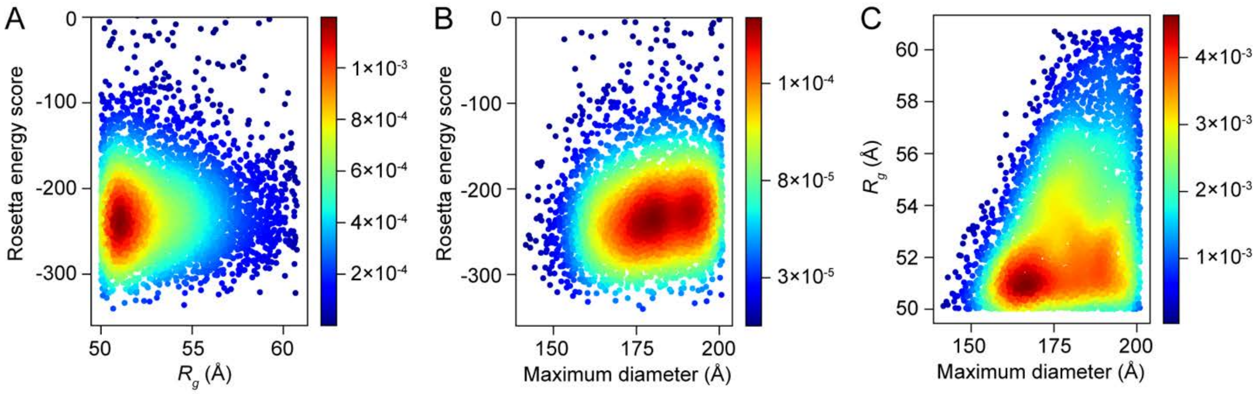

Figure S6. Filtering Rosetta BRD4 tandem bromodomain models by SAXS constraints.

(A) Rosetta models were constrained to $D_{max} \leq 201$ Å and 49.9 Å $\leq R_g \leq$ 59.9 Å. Rosetta energy score convergence was not observed across allowed **(B)** R_g or **(C)** D_{max} ranges. Color bars represent kernel density estimation of the probability density functions for each distribution calculated using the gaussian_kde method provided by the scipy.stats Python class.

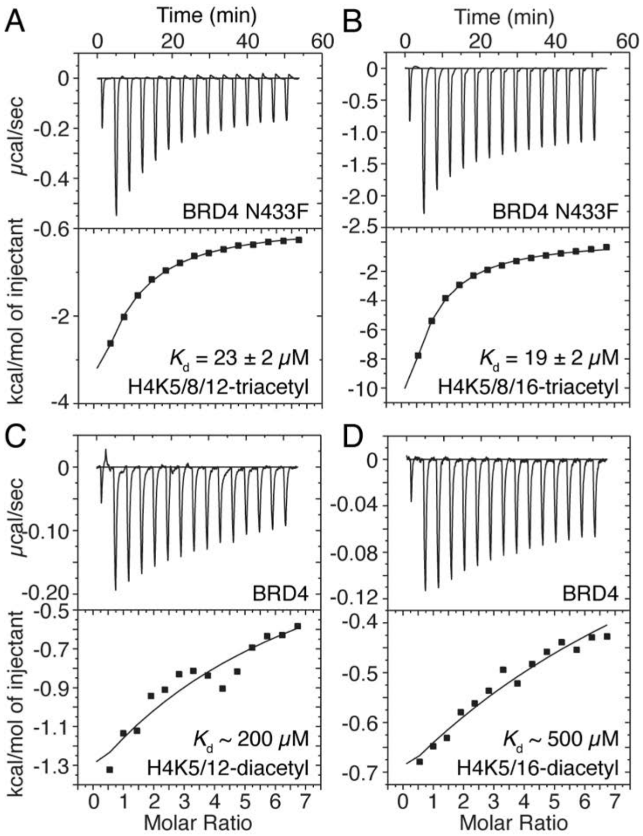

Figure S7. BRD4-BD1 binding to the H4K5/8-diacetyl histone modification is the primary mediator of BRD4 affinity toward acetylated histone H4 tails. ITC traces (n=1) of tandem BRD4 N433F (aa 36-460) binding to **(A)** H4K5/8/12-triacetyl and **(B)** H4K5/8/16-triacetyl histone peptides. ITC traces (n=1) of tandem BRD4 wild-type (aa 36-460) binding to **(C)** H4K5/12-diacetyl and **(D)** H4K5/16-diacetyl histone peptides.

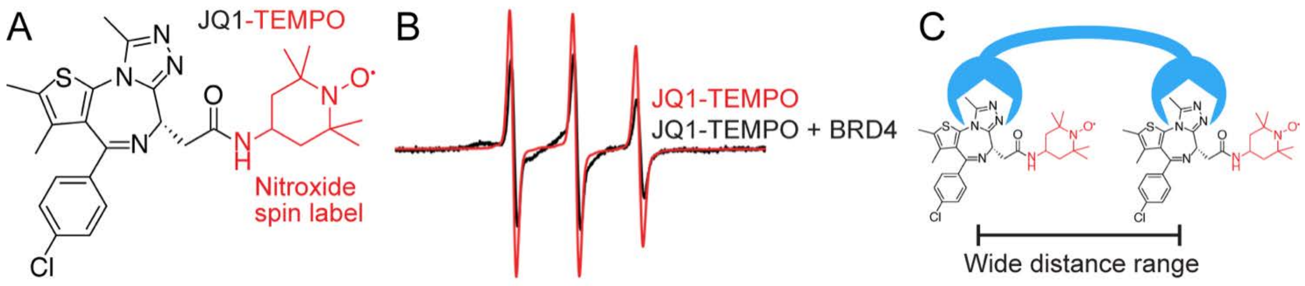

Figure S8. EPR-based strategy for BRD4 tandem bromodomain inter-acetyl-lysine binding site measurements. **(A)** Structure of JQ1-TEMPO EPR probe. **(B)** Continuous wave (CW) EPR detection of JQ1-TEMPO binding to the BRD4 tandem bromodomains. **(C)** No double electron-electron resonance (DEER) signal was observed in the JQ1-TEMPO + BRD4 tandem bromodomain sample, indicating the bromodomains of the tandem BRD4 construct do not consistently adopt any discrete distances that result in unique and detectable DEER signals.

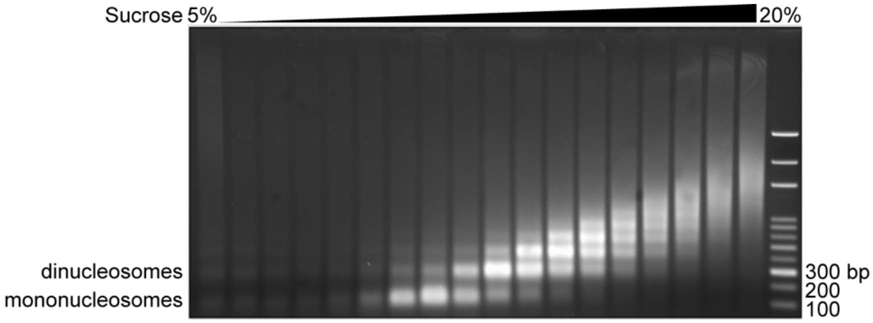

Figure S9. Sucrose gradient purification of calf thymus nucleosomes. Fractions containing primarily mononucleosomes were combined and used in subsequent BRD4 binding assays.

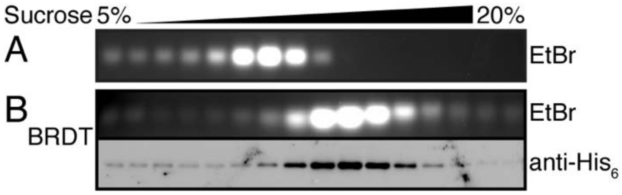

Figure S10. Sucrose gradient binding assays of BRDT tandem bromodomains. **(A)** Control sucrose gradient demonstrates the sedimentation rate of calf thymus mononucleosomes without bromodomain-containing proteins. **(B)** Sucrose gradient binding assays show that BRDT tandem bromodomains physically associate with calf thymus mononucleosomes and increase their sedimentation rates relative to control.

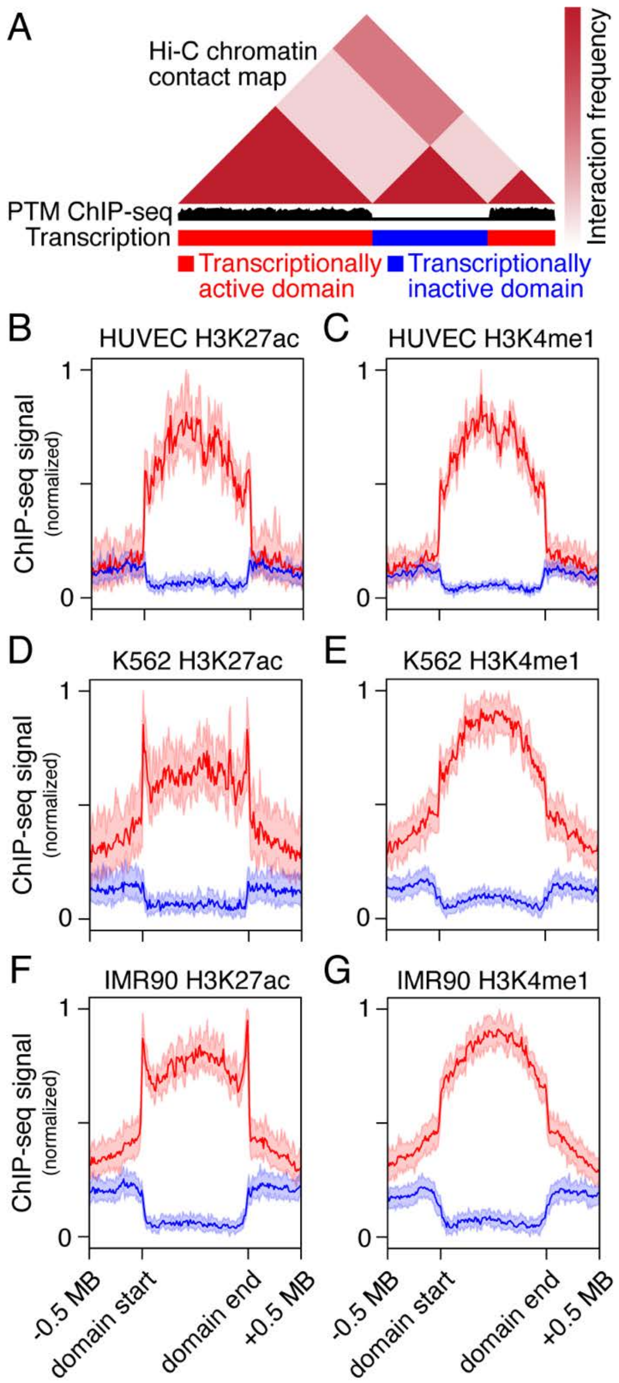

Figure S11. K-means clustering of H3K27ac and H3K4me1 ChIP-seq profiles across annotated compartmental domains (GSE63525). **(A)** Schematic depicting transcriptionally active (red, based on H3K27ac ChIP-seq signal clustering) and transcriptionally inactive (blue, based on H3K4me1 ChIP-seq signal clustering) compartmental domain designations. Average H3K27ac and H3K4me1 ChIP-seq signals are plotted across compartmental domains for **(B-C)** IMR90, **(D-E)** HUVEC, and **(F-G)** K562 cell lines, where high occupancy is denoted by red traces and low occupancy is denoted by blue traces.

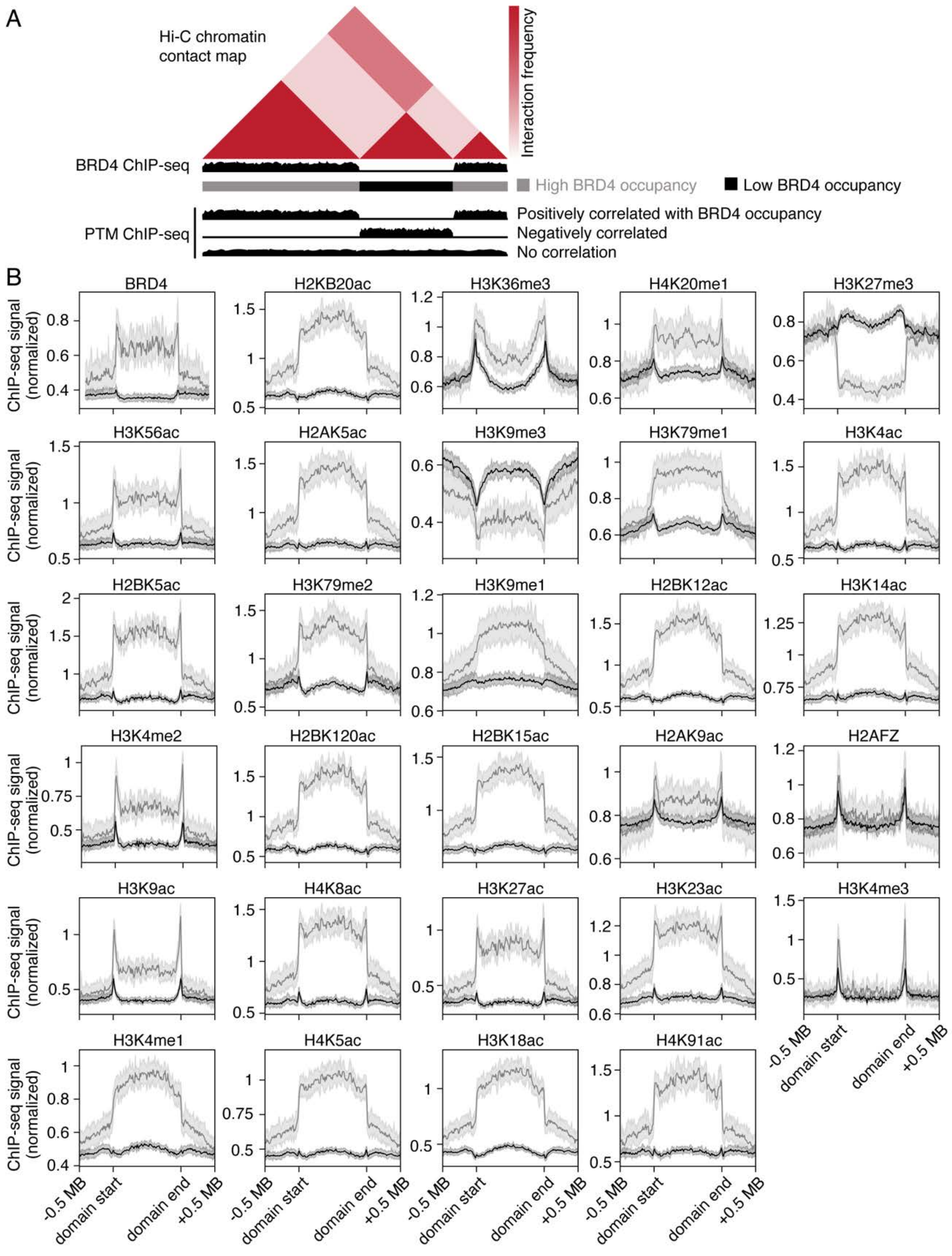

Figure S12. Association between BRD4 ChIP-seq signal and epigenetic histone PTMs across IMR90 compartmental domains. **(A)** Schematic depicting high and low BRD4 occupancy designations based on BRD4 ChIP-seq signal clustering. **(B)** ChIP-seq profiles for 28 histone post-translational modifications calculated across experimental domains in regions of high (gray) and low (black) BRD4-occupancy.

Table S1. Values calculated from SAXS analysis of tandem BET bromodomains.

**Protein**	**R_g (Å)**	**D_{max} (Å)**
BRDT	53.8 ± 1.2	188 ± 4
BRD2	48.6 ± 2.9	181 ± 8
BRD3	49.1 ± 2.6	183 ± 4
BRD4	55.4 ± 5.5	200 ± 1

Table S2. R_g values calculated by P(r) analysis vs. Guinier analysis of tandem BET bromodomains.

**Protein**	**R_g P(r) (Å)**	**R_g Guinier (Å)**
BRDT	53.8 ± 1.2	50.9 ± 4.5
BRD2	48.6 ± 2.9	49.6 ± 3.0
BRD3	49.1 ± 2.6	49.7 ± 5.2
BRD4	54.2 ± 5.1	58.4 ± 11.0

References

1. Bradford MM. A rapid and sensitive method for the quantitation of microgram quantities of protein utilizing the principle of protein-dye binding. Analytical Biochemistry. 1976;72(1):248-54. doi: 10.1016/0003-2697(76)90527-3. PubMed PMID: 942051.
